# Role of Interleukin-6 in the Antigen-Specific Mucosal Immunoglobulin A Responses Induced by CpG Oligodeoxynucleotide-Loaded Cationic Liposomes

**DOI:** 10.3390/membranes12060635

**Published:** 2022-06-20

**Authors:** Rui Tada, Emi Honjo, Shoko Muto, Noriko Takayama, Hiroshi Kiyono, Jun Kunisawa, Yoichi Negishi

**Affiliations:** 1 Department of Drug Delivery and Molecular Biopharmaceutics, School of Pharmacy, Tokyo University of Pharmacy and Life Sciences, Tokyo 192-0392, Japan; emi0228_shizuku@nifty.com (E.H.); shoko.muto@gmail.com (S.M.); nori-k0123_suns-@hotmail.co.jp (N.T.); negishi@toyaku.ac.jp (Y.N.); 2 Division of Mucosal Immunology and International Research and Development Center for Mucosal Vaccines, Department of Microbiology and Immunology, The Institute of Medical Science, The University of Tokyo, Tokyo 108-8639, Japan; kiyono@ims.u-tokyo.ac.jp (H.K.); kunisawa@nibiohn.go.jp (J.K.); 3 Laboratory of Vaccine Materials, Center for Vaccine and Adjuvant Research and Laboratory of Gut Environmental System, National Institutes of Biomedical Innovation, Health and Nutrition (NIBIOHN), Osaka 567-0085, Japan

**Keywords:** CpG ODN, cationic liposome, interleukin-6, mucosal vaccine, mucosal adjuvant

## Abstract

An advantage of mucosal vaccines over conventional parenteral vaccines is that they can induce protective immune responses not only at mucosal surfaces but also in systemic compartments. Despite this advantage, few live attenuated or inactivated mucosal vaccines have been developed and applied clinically. We recently showed that the intranasal immunization of ovalbumin (OVA) with class B synthetic oligodeoxynucleotides (ODNs) containing immunostimulatory CpG motif (CpG ODN)-loaded cationic liposomes synergistically exerted both antigen-specific mucosal immunoglobulin A (IgA) and systemic immunoglobulin G (IgG) responses in mice. However, the mechanism underlying the mucosal adjuvant activity of CpG ODN-loaded liposomes remains unknown. In the present study, we showed that the intranasal administration of CpG ODN-loaded cationic liposomes elicited interleukin (IL)-6 release in nasal tissues. Additionally, pre-treatment with an anti-IL-6 receptor (IL-6R) antibody attenuated antigen-specific nasal IgA production but not serum IgG responses. Furthermore, the intranasal administration of OVA and CpG ODN-loaded cationic liposomes increased the number of IgA^+^/CD138^+^ plasma cells and IgA^+^/B220^+^ B cells in the nasal passages. This increase was markedly suppressed by pre-treatment with anti-IL-6R blocking antibody. In conclusion, IL-6 released by CpG ODN-loaded cationic liposomes at the site of administration may play a role in the induction of antigen-specific IgA responses by promoting differentiation into IgA^+^ plasma cells for IgA secretion from B cells.

## 1. Introduction

Infectious diseases are a major cause of death worldwide and continue to pose a grave threat to human health, despite considerable advancements made in the medical field [1,2]. This is evident from the outbreak of the coronavirus disease 2019 (COVID-19), caused by severe acute respiratory syndrome-coronavirus 2 (SARS-CoV-2) in 2019, which has had a tremendous impact on our society [3]. Therefore, it is imperative to continue research and develop new antibiotics and vaccines to combat infectious diseases.

Vaccines are considered to be one of the greatest inventions in human history and have protected humankind from illness and death due to infectious diseases. Recent advances in science and technology have led to the launch of a series of next-generation vaccines, including two RNA vaccines [3,4], one viral vector vaccine [5], and one DNA vaccine [6]. These next-generation vaccines are highly efficacious; however, their ineffectiveness in preventing infection, as well as the transmission to others, is an issue that needs to be tackled. Mucosal vaccines are anticipated to be the next-generation vaccines that can effectively address these shortcomings.

The mucosal vaccines that have recently emerged are superior to conventional parenteral vaccines, as they can induce protective immune responses in the systemic compartments as well as at mucosal surfaces, which are sites of invasion and colonization for most pathogens [7,8]. Although live-attenuated and inactivated mucosal vaccines offer many advantages over conventional vaccines, only a few have been implemented in clinical settings [9]. Both live-attenuated and inactivated vaccines contain the pathogen. Consequently, unexpected side effects due to toxicity or antigenicity are inevitable. Therefore, it is desirable to develop subunit mucosal vaccines using pathogen-derived antigens. Nonetheless, no subunit mucosal vaccine has been approved for human use in clinics, mainly due to the lack of safe and effective mucosal adjuvants [10,11].

We have recently established that cationic liposomes composed of 1,2-Dioleoyl-3-trimethylammonium-propane (DOTAP) and β-[*N*-(*N*′,*N*′-dimethylaminoethane)-carbamoyl] (DC-chol) act as a mucosal adjuvant for nasal vaccine formulations. During the course of our study, we have found that the positive surface charge of the liposome membrane is crucial for adjuvant activity [12]. In addition, we also demonstrated that the intranasal immunization of a model antigen, namely ovalbumin (OVA), with class B synthetic oligodeoxynucleotides (ODNs) containing immunostimulatory CpG motif (CpG ODN)-loaded cationic liposomes synergistically exerted both antigen-specific mucosal immunoglobulin A (IgA) and systemic immunoglobulin G (IgG) responses in mice [13]. However, the mechanism underlying the mucosal adjuvant activity of CpG ODN-loaded liposomes remains unknown. Cytokines play a role in the initiation of a wide range of immune responses, including the induction of mucosal immune responses to foreign antigens. For instance, interleukin (IL)-6 exerts a pleiotropic effect on various immune responses [14], including the induction of IgA class switch recombination (CSR), which is required to produce IgA in the mucosa [15]. In the present study, we hypothesized that IL-6 is associated with the induction of mucosal immune responses of CpG ODN-loaded cationic liposomes when administered nasally to mice. Therefore, we explored the involvement of IL-6 in CpG ODN-loaded cationic liposome-induced antigen-specific antibody responses in mice.

## 2. Materials and Methods

### 2.1. Animals

BALB/cCrSlc female mice (6 weeks old) were obtained from Japan SLC (Shizuoka, Japan). All mice were housed in a specific pathogen-free environment; they were used at 7–9 weeks of age in the experiments. The protocols for the animal experiments were approved by the Committee for Laboratory Animal Experiments at the Tokyo University of Pharmacy and Life Sciences.

### 2.2. Materials

1,2-Dioleoyl-3-trimethylammonium-propane (DOTAP) was purchased from Avanti Polar Lipids (Alabaster, AL, USA). Additionally, 3β-[*N*-(*N*′,*N*′-dimethylaminoethane)-carbamoyl] (DC-chol) and egg white OVA were obtained from Sigma-Aldrich (St. Louis, MO, USA). Class B CpG ODN 1668 (5′-tccatgacgttcctgatgct-3′; small letters indicate phosphorothioate linkage) was synthesized by Sigma-Aldrich (Woodlands, TX, USA). Endotoxin-free phosphate-buffered saline (PBS) was acquired from FUJIFILM Wako Pure Chemical Industries (Osaka, Japan). Rat anti-mouse IL-6 receptor (IL-6R) antibody (clone MR16-1) was provided by Chugai Pharmaceutical Co., Ltd. The corresponding rat IgG1 k isotype control monoclonal antibody (clone RTK2071) for MR16-1 was purchased from BioLegend (San Diego, CA, USA).

### 2.3. Preparation of CpG ODN-Loaded Cationic Liposomes

Liposomes were prepared as described previously [12,16]. Briefly, 10 μmol of total lipids (DOTAP:DC-chol at 1:1 mol ratios) was mixed in a glass tube and evaporated to dryness in vacuo. Retained lipid films were hydrated by adding 250 μL of PBS and vortexing for 5 min. Multilamellar vesicles were then extruded 10 times through a 100 nm pore polycarbonate membrane (ADVANTEC, Tokyo, Japan) and sterilized using 0.45 μm filter membranes (IWAKI, Tokyo, Japan). Ten micrograms of CpG ODN solution dissolved in PBS was added to 200 nmol of prepared cationic liposomes by vortexing and then were incubated for 5 min at 25 °C. After incubation, CpG ODN-loaded cationic liposomes were used immediately for the assessment of their mucosal adjuvant activities, as described below.

### 2.4. Immunization Schedule and Blocking Effect of Anti-IL-6R Antibody on the Mucosal Adjuvanticity of CpG ODN-Loaded Cationic Liposomes

BALB/cCrSlc female mice were pre-treated with anti-IL-6R antibody or isotype control antibody (250 µg/mouse) two days prior to the first immunization (day-2) and 1 h prior to each subsequent immunization (days 0 and 7), as described in an earlier study [17]. The mice were nasally immunized with PBS (vehicle) or OVA (5 µg/mouse) with CpG ODN-loaded cationic liposomes (10 µg as CpG ODN/mouse) at a volume of 13 µL on days 0 and 7 under anesthesia with an intraperitoneal injection of 0.2 mL of a mixture containing 0.75 mg/kg of medetomidine, 4 mg/kg of midazolam, and 5 mg/kg of butorphanol tartrate. After sacrificing, serum and nasal wash samples were collected on day 14. To assess systemic OVA-specific antibody responses, blood was collected and incubated at 25 °C for 30 min. Subsequently, the obtained samples were incubated for 1 h at 4 °C, and the serum samples were collected after centrifugation at 1200× *g* for 30 min. To monitor the induction of OVA-specific IgA in nasal washes, nasal wash samples (250 µL of cold PBS) were collected [18,19]. The samples were stored at –80 °C until further analysis.

### 2.5. Expression of IL-6 and Transforming Growth Factor-Beta (TGF-β) in Leukocytes

Nasal-associated lymphoid tissue (NALT) and nasal passages were prepared as previously described [20,21]. Mice that were nasally immunized with CpG ODN-loaded cationic liposomes (10 µg CpG ODN/mouse), OVA (5 µg/mouse), or both (10 µg CpG ODN and 5 µg OVA/mouse) were sacrificed by cervical dislocation, and their NALTs and nasal passages were harvested. After treatment with ammonium chloride–potassium (ACK) lysis buffer to destroy red blood cells, the cells were subjected to treatment with 250 µL of a lysis buffer (10 mM Tris-HCl (pH 7.2) containing 50 mM sodium chloride (NaCl), 1% Triton X-100, 0.1 mM sodium orthovanadate (Na_3_VO_4_), 1 mM phenylmethylsulfonyl fluoride (PMSF), 5 mM ethylenediaminetetraacetic acid (EDTA), and 10 µg/mL aprotinin, and 10 µg/mL leupeptin). After centrifugation (1500× *g*, 4 °C, 5 min), the supernatants were collected and stored at −80 °C until further assays.

### 2.6. Cytokine Assay

The IL-6 concentration in the sample was determined using ELISA MAX^TM^ Standard Sets (BioLegend), according to the manufacturer’s instructions. The TGF-β concentration in the samples was determined using the TGF-β1 Human/Mouse Uncoated ELISA Kit (Invitrogen, Waltham, MA, USA). Data are presented as the mean ± standard deviation from biological triplicates.

### 2.7. Evaluation of OVA-Specific Antibody by Enzyme-Linked Immunosorbent Assay (ELISA)

OVA-specific antibody titers were evaluated by ELISA, as described earlier [20]. Briefly, a 96-well Nunc MaxiSorp plate (Thermo Scientific, Waltham, MA, USA) was coated with 1.25 μg of OVA (Sigma-Aldrich, St. Louis, MO, USA) in 0.1 M carbonate buffer (pH 9.5) overnight at 4 °C. The plate was then washed with PBS containing 0.05% Tween 20 (PBST) and blocked with 1% bovine serum albumin (BSA; FUJIFILM Wako Pure Chemical Industries) containing PBST (BPBST) at 37 °C for 60 min. The plates were washed with PBST and treated with biotin-conjugated anti-mouse IgA, IgG1, or IgG2a secondary antibodies (BioLegend) in BPBST. Following this, avidin–horseradish peroxidase (HRP) (BioLegend) in BPBST was added to each well. After washing with PBST, the plates were color developed using a 3,3′,5,5′-tetramethylbenzidine (TMB) substrate system (KPL, Gaithersburg, MD, USA). Color development was terminated using 1 N phosphoric acid, and the optical density was measured at 450 nm (reference filter 650 nm) using a Varioskan Flash Micro Plate Reader (Thermo Scientific).

### 2.8. Cell Isolation and Flow Cytometric Analysis

A week after the last immunization, the NALT and nasal passage were harvested from the upper jaw of the mice. Mononuclear cells from NALT were obtained by scraping the NALT or nasal passage tissue. After treatment with ACK lysis buffer to remove red blood cells, the cells were suspended in a staining buffer (PBS containing 2% heat-inactivated fetal bovine serum and 0.1% sodium azide) and then treated with anti-mouse CD16/32 antibody (clone 93; BioLegend) for 30 min at 4 °C to block Fc receptors. After washing with staining buffer, the cells were stained with phycoerythrin (PE)-conjugated rat anti-mouse B220 (clone RA3-6B2; BD Bioscience, San Diego, CA, USA), fluorescein isothiocyanate (FITC)-conjugated rat anti-mouse IgA (clone C10-3; BD Bioscience), or PE-conjugated rat anti-mouse CD138 (clone 281-2; BD Bioscience) for 30 min at 4 °C. After washing with staining buffer, the cells were incubated with 7-amino-actinomycin D (7-AAD; BioLegend) for 10 min at 4 °C and then analyzed using a FACSCalibur instrument (BD Bioscience).

### 2.9. Statistics

Statistical analyses were performed using a *t*-test with Welch’s correction or the Mann–Whitney U test, calculated by GraphPad Prism 8 (GraphPad Software, San Diego, CA, USA).

## 3. Results

### 3.1. IL-6 Expression in the Nasal Mucosa of Mice Nasally Administered CpG ODN-Loaded Cationic Liposomes

**Figure 1:**
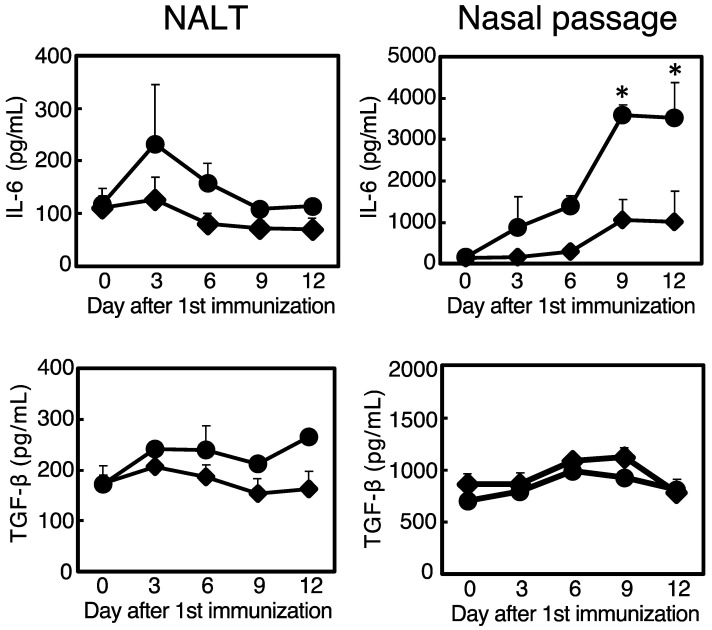
Production of interleukin (IL)-6 and transforming growth factor-beta (TGF-β) in nasal-associated lymphoid tissues (NALTs) and nasal passages from mice nasally immunized with CpG oligodeoxynucleotide (ODN)-loaded cationic liposomes in combination with ovalbumin (OVA) (◆), or CpG ODN in combination with OVA (●). NALTs and nasal passages were harvested 0, 3, 6, 9, or 12 d after immunization. After extraction, the levels of IL-6 and TGF-β were analyzed using an enzyme-linked immunosorbent assay (ELISA). Cytokine levels represent the mean ± standard deviation of biological triplicates. CpG ODN plus OVA, *n* = 3; CpG ODN-loaded cationic liposomes plus OVA, *n* = 3. Significant differences were calculated using an unpaired *t*-test with Welch’s correction. * *p* < 0.05.

Antigen-specific immune responses are regulated by a variety of host humoral factors, such as cytokines [22,23,24]. For example, antigen-specific IgA production is regulated by IL-6 and TGF-β [25,26]. To investigate the mode of action(s) of mucosal adjuvanticity of CpG ODN-loaded cationic liposomes, we tested the production of IL-6 and TGF-β, which can augment antigen-specific mucosal IgA responses, after nasally administering CpG ODN-loaded cationic liposomes. Accordingly, the intranasal administration of CpG ODN-loaded cationic liposomes induced the production of IL-6 in nasal passages as early as 3 days post-administration, peaking at 9 days post-administration; however, this was not observed in the NALT. In contrast, the intranasal administration of CpG ODN-loaded cationic liposomes did not induce TGF-β production in either the NALT or nasal passage (Figure 1). Together, these data demonstrated that CpG ODN-loaded cationic liposomes are potent inducers of IL-6 in the nasal mucosa during nasal administration.

### 3.2. Anti-IL-6R Blocking Antibody Ameliorated Antigen-Specific Mucosal IgA, but Not Systemic IgG, by CpG ODN-Loaded Cationic Liposomes

**Figure 2:**
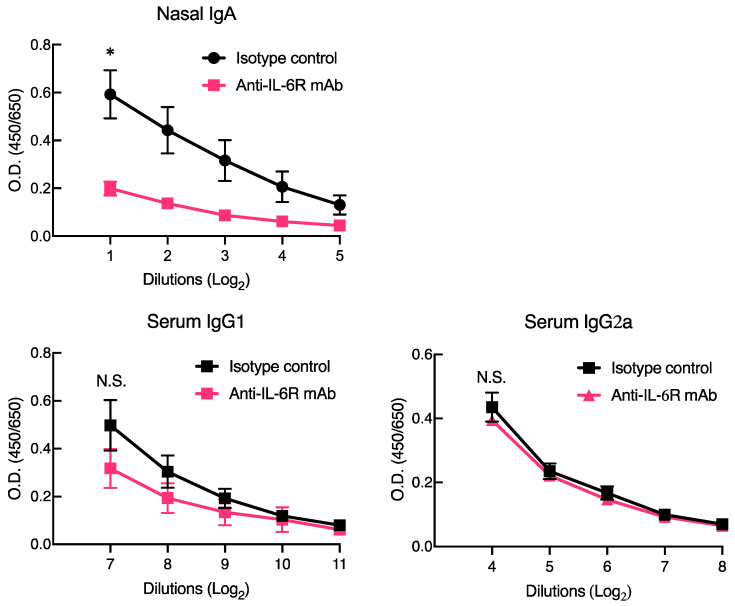
Inhibitory effect of anti-interleukin-6 receptor (IL-6R) blocking antibodies on CpG ODN-loaded cationic liposome-induced OVA-specific antibody responses. BALB/c mice were intraperitoneally treated with anti-IL-6R antibody (250 µg/mouse) or the respective isotype control antibody (250 µg/mouse) two days prior to the first immunization (day-2) and 1 h prior to each subsequent immunization (days 0 and 7). Mice were then immunized intranasally with OVA (5 µg/mouse) in combination with CpG ODN-loaded cationic liposomes (10 µg/mouse as CpG ODN) at a volume of 13 µL twice weekly. One week after the last immunization, nasal washes and serum samples were collected. OVA-specific antibody responses were tested using ELISA. Isotype control, *n* = 4; anti-IL-6R mAb, *n* = 4. Significant differences were evaluated using the Mann–Whitney U test. * *p* < 0.05.

The above experiment indicated the possible involvement of IL-6 in the mucosal adjuvanticity of CpG ODN-loaded cationic liposomes. To assess the role of IL-6 in mucosal adjuvant activity, we intranasally immunized mice with OVA and CpG ODN-loaded cationic liposomes that were pre-treated with anti-IL-6R antibody, which has been reported to block the biological activities of IL-6 in vivo [27,28,29]. Pre-treatment with anti-IL-6R antibody clearly impaired OVA-specific nasal IgA production in mice that were nasally immunized with OVA in combination with CpG ODN-loaded cationic liposomes when compared to pre-treatment with isotype control antibody (Figure 2). Conversely, no difference was observed in the production of OVA-specific serum IgG1 and IgG2a (Figure 2). The obtained data suggested that the IgA responses in mucosal compartments exerted by the intranasal immunization of CpG ODN-loaded cationic liposomes depend on IL-6 but not IgG responses in the body.

### 3.3. Role of IL-6 in the Differentiation into IgA-Secreting Plasma Cells

**Figure 3:**
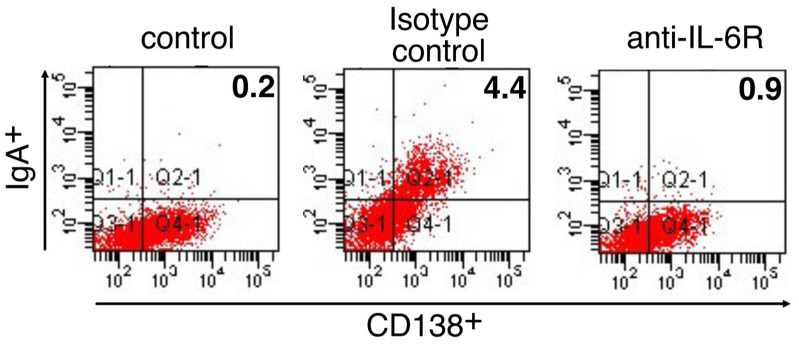
Influence of anti-IL-6R blocking antibodies on the differentiation into IgA^+^ plasma cells in the nasal passage induced by CpG ODN-loaded cationic liposomes. BALB/c mice were intraperitoneally treated with anti-IL-6R antibody (250 µg/mouse) or the respective isotype control antibody (250 µg/mouse) two days prior to the first immunization (day-2) and 1 h prior to each subsequent immunization (days 0 and 7). Mice were then immunized intranasally with PBS or OVA (5 µg/mouse) in combination with CpG ODN-loaded cationic liposomes (10 µg/mouse as CpG ODN) at a volume of 13 µL twice weekly. A week after the last immunization, IgA^+^ plasma cells in the nasal passage were analyzed by flow cytometry. Control, *n* = 4; isotype control, *n* = 4; anti-IL-6R mAb, *n* = 4.

**Figure 4:**
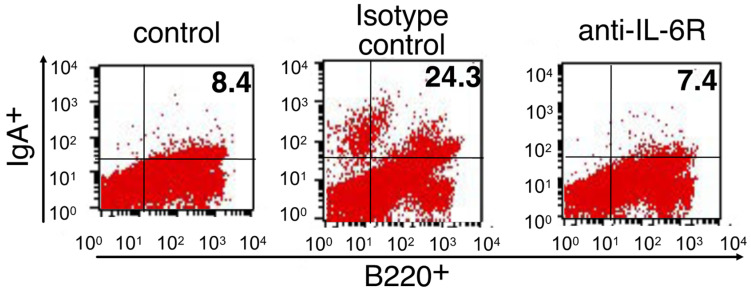
Influence of anti-IL-6R blocking antibodies on the differentiation into IgA^+^ B cells in the nasal passage induced by CpG ODN-loaded cationic liposomes. BALB/c mice were intraperitoneally treated with anti-IL-6R antibody (250 µg/mouse) or the respective isotype control antibody (250 µg/mouse) two days prior to the first immunization (day-2) and 1 h prior to each subsequent immunization (days 0 and 7). Mice were then immunized intranasally with PBS or OVA (5 µg/mouse) in combination with CpG ODN-loaded cationic liposomes (10 µg/mouse as CpG ODN) at a volume of 13 µL twice weekly. A week after the last immunization, IgA^+^ B cells in the nasal passage were analyzed by flow cytometry. Control, *n* = 4; isotype control, *n* = 4; anti-IL-6R mAb, *n* = 4.

CSR to IgA of B cells by antigen exposure in combination with diverse stimuli generally occurs at the induction site, NALT, in the nasal mucosa. IgA^+^ B cells then migrate to the effector site, the nasal passage, which is the site of differentiation into IgA-secreting plasma cells [30]. Therefore, we next examined the role of IL-6 in these processes induced by CpG ODN-loaded cationic liposomes. As shown in Figure 3, the intranasal administration of OVA and CpG ODN-loaded cationic liposomes increased the number of IgA^+^/CD138^+^ plasma cells in nasal passages. This increase was markedly suppressed by pre-treatment with anti-IL-6R blocking antibody. Moreover, the intranasal administration of OVA and CpG ODN-loaded cationic liposomes increased the number of IgA^+^/B220^+^ B cells within the nasal passages. This increase was diminished upon pre-treatment with anti-IL-6R blocking antibody (Figure 4). Taken together, these data suggested that IL-6 induced by CpG ODN-loaded cationic liposomes at the site of administration may play a role in the induction of antigen-specific IgA responses by promoting differentiation into IgA^+^ plasma cells for IgA secretion from B cells.

## 4. Discussion

With the emergence of the COVID-19 pandemic, the social demand for vaccine development has increased substantially. To meet these demands, research and development on vaccine platforms, with a wide range of modalities, is of paramount importance. Among these, mucosal vaccine platforms are considered to be the most favorable because of their ability to induce antigen-specific immune responses at mucosal surfaces, where they act as the entry and colonization sites for most pathogens [7,8,31]. In this context, we have been searching for mucosal adjuvants for nasal vaccines that are pivotal for developing subunit types of mucosal vaccine formulations. In the course of our study, we found that CpG ODN-loaded cationic liposomes showed mucosal adjuvant activity in mice [13]. However, the molecular mechanisms by which CpG ODN-loaded cationic liposomes enhance antigen-specific mucosal immune responses remain unknown.

Understanding the molecular mechanism(s) by which mucosal adjuvants enhance antigen-specific immunity is needed for the development of safe and effective mucosal vaccine formulations. The physicochemical properties of liposomes, such as the surface charge of the membrane, are known to affect their adjuvant activities. For instance, we have previously found that only cationic liposomes induced antigen-specific antibody production [12]. In recent years, the role of activating the innate immune response in adjuvanticity has received much attention rather than antigen delivery to antigen-presenting cells (APCs). For instance, certain adjuvants cause tissue damage locally, followed by cell death, resulting in the release of molecules outside cells that are normally localized only inside cells. These molecules, termed damage-associated molecular patterns (DAMPs), trigger innate immune responses via recognition by pattern-recognition receptors (PRRs) [32]. The resultant activation of innate immunity induces the subsequent release of various cytokines, initiating acquired immune responses, such as antibody responses toward antigens [33]. As such, IL-6 is an inflammatory cytokine involved in shaping adaptive immunity through the induction of IgA CSR and facilitating differentiation into IgA-secreting cells in the mucosa [14,15].

Herein, we revealed that CpG ODN-loaded liposomes trigger IL-6 expression at the site of administration (Figure 1). This result can be considered demonstrative of the additive or synergistic effects of CpG ODN and cationic liposomes, as we previously reported that the cationic liposomes themselves are potent inducers of IL-6 expression. Pre-treatment with anti-IL-6R antibody to block IL-6 signaling debilitated antigen-specific nasal IgA production but not antigen-specific serum IgG production (Figure 2). Furthermore, the intranasal administration of OVA and CpG ODN-loaded cationic liposomes increased the number of IgA^+^/CD138^+^ plasma cells (Figure 3) and IgA^+^/B220^+^ B cells (Figure 4) in nasal passages. This increase was markedly suppressed by pre-treatment with anti-IL-6R blocking antibody (Figure 3 and Figure 4); consequently, it was inferred that IL-6 induced by CpG ODN-loaded cationic liposomes at the site of administration would play a role in the induction of antigen-specific IgA responses by promoting differentiation into IgA^+^ plasma cells for IgA secretion from B cells. However, the specific cell types that produce IL-6, and the underlying governing mechanisms, remain unknown. Greene et al. reported that the adjuvant activity of type II heat-labile enterotoxin (LT-IIb) induces the chemotaxis of IL-6-expressing neutrophils to the site of administration. Furthermore, as adjuvant activity was diminished in IL-6 knockout mice, the adjuvant activity of LT-IIb is dependent on IL-6-producing neutrophils, which migrate upon the administration of the adjuvant [34]. The limitation of this study is that CpG ODN-loaded cationic liposomes induced the production of IL-6 in the nasal mucosa. The identification of cell types induced by this adjuvant system and IL-6 expression is currently under investigation. However, they reportedly induce the expression of IL-6 in lymphocytes, monocytes, macrophages, and dendritic cells via recognition by TLR9 [35]. Therefore, further investigation is required to understand the molecular mechanism(s) involved.

In conclusion, we elucidated the mechanisms by which antigen-specific mucosal IgA responses are enhanced following the intranasal administration of CpG ODN-loaded cationic liposomes with antigenic proteins. Although further experiments are needed to completely understand the molecular mechanism(s) underlying mucosal adjuvanticity, we believe that nasal vaccine systems utilizing CpG ODN-loaded cationic liposomes could prove useful in treating infectious diseases in the future. Authors should discuss the results and how they can be interpreted from the perspective of previous studies and of the working hypotheses. The findings and their implications should be discussed in the broadest context possible. Future research directions may also be highlighted.

## Data Availability

Data is contained within the article.
